# Neonatal seizures, experience at Children Hospital and Institute of Child Health Multan

**DOI:** 10.12669/pjms.295.3847

**Published:** 2013

**Authors:** Abdur Rehman Malik, Ahmed Iqbal Quddusi, Naila

**Affiliations:** 1 Dr. Abdur Rehman Malik, MBBS, DCH, FCPS (Pediatrics), Senior Registrar, Department of Neonatology, Children Hospital and Institute of Child Health, Multan, Pakistan.; 2 Dr. Ahmed Iqbal Quddusi, MBBS, FCPS (Pediatrics), Assistant Professor and Head of Department of Neonatology,Children Hospital and Institute of Child Health, Multan.; 3 Dr. Naila, MBBS FCPS (Pediatrics),Senior Registrar, Department of Neonatology, Children Hospital and Institute of Child Health, Multan, Pakistan.

**Keywords:** Neonates, Seizures, Etiology

## Abstract

***Objective:*** The study was conducted to determine the etiology of seizures in neonates.

***Methods:*** This was a descriptive Cross-sectional study. This study was conducted at neonatal unit of children hospital and institute of child health Multan from June 2012 to April 2013. A total of 285 neonates who presented with seizures were enrolled for the possible causes. First line investigations including blood glucose, serum calcium, serum magnesium, serum sodium, complete blood counts, serum creatinine, liver function tests, ABGs, cerebrospinal fluid examination, cranial ultrasound and EEG were done in all neonates. Second line investigations (blood culture, CT scan, MRI, screening for inborn error of metabolism and Torch antibody titer) were done in selected neonates. The data was analyzed using SPSS-10.

***Results:*** Out of 285 neonates, 175(61.4%) were male and 110(38.6%) female. There were 199(69.8%) term and 86 (30.2%) preterm. Birth asphyxia was the commonest cause of seizures and present in 53.7% neonates and metabolic disturbance in 17.5%. Seizures with unknown etiology were present in 5.3% neonates.

***Conclusion:*** In most of the cases, the causes of neonatal seizures were present. Birth asphyxia was the main etiology identified. However, to establish the exact cause of seizures, more extensive work up is needed.

## INTRODUCTION

Seizures are more common in the neonatal period than in any other stage and affects approximately 1% of all neonates.^1^ A seizure is defined clinically as a paroxysmal alteration in neurologic function, i.e. motor, behavior and/or autonomic function. This definition includes, epileptic seizures (phenomena associated with corresponding EEG seizure activity e.g. clonic seizures), non-epileptic seizures (clinical seizures without corresponding EEG correlate e.g. subtle and generalized tonic seizures) and EEG seizures (abnormal EEG activity with no clinical correlation).^2^ Historically, neonatal seizures are divided into subtle, clonic, tonic and myoclonic seizures. Clonic and tonic seizures are further classified into focal and generalized types.^3^ As compared to older children; generalized tonic-clonic seizures are rare in neonates.^4^

Determination of etiology is critical, both for treatment and disease prognosis.^5^ Birth asphyxia is the commonest cause of neonatal seizures.^3^ Other causes include septicemia with or without CNS infection, transient metabolic disorder (hypoglycemia, hypocalcaemia, hypomagnesemia and hypernatremia.^6^ Hypocalcaemia is the commonest metabolic disorder followed by hypoglycemia), intracranial bleed, brain malformation, inborn errors of metabolism (urea cycles defects, mitochondrial abnormalities), tetanus, kernicterus and pyridoxine dependency.^6^^,^^7^ Other causes include polycythemia, maternal narcotic withdrawal, drug toxicity (e.g. theophylline, doxapram), local anesthetic (lignocaine toxicity) injection into scalp and phacomatosis (e.g. tuberous sclerosis, incontinentia pigmentii).^8^^,^^9^

Outcome is predicted by the underlying aetiology.^9^ Patients with hypoxic ischemic encephalopathy (HIE), intra-ventricular hemorrhage and structural brain malformation have the worst prognosis,^9^^,^^10^ while those with transient metabolic abnormalities and benign idiopathic or familial etiologies have the best prognosis.^11^ Overall 10–50% of patients die and 50% develop long term complications like epilepsy, cerebral palsy and mental retardation.^8^ The present study was planned to evaluate the different causes of neonatal seizures in our setup to have an effective strategy for their management and prognosis.

## METHODS

This prospective descriptive study was done at the neonatal unit of children hospital and institute of child health Multan from June 2012 to April 2013. A total of 285 neonates of either gender admitted in neonatal unit through paediatrics OPD and emergency room, who presented with seizures, were included. An informed written consent from parents/attendants was obtained and benefits of the study were explained. The approval of ethical committee of Children Hospital and Institute of Child Health (Department of Neonatology), Multan was taken for this study.

Jitteriness or sleep-related muscular activities and other non-seizures movements were differentiated from seizures on the basis of absence orbito-occular movements, absence of autonomic changes, normal sensorium and cessation of movements on holding the affected parts and confirmed by EEG changes.

Detailed history including age of the neonates, duration and type of seizures, antenatal history of intrauterine infection (fever, rash, lymphadenopathy), maternal diabetes, maternal hypertension, maternal endocrine disorders and maternal drug intake during pregnancy were taken. Delivery details were also taken including place of delivery (home, private clinic, hospital), mode of delivery (spontaneous vaginal, instrumental, c-section), duration of labour, delivery attendants (doctor, dai, LHV), history of resuscitation, and APGAR score <3 at 1 and 5 minutes of age. Family history of neonatal fits, deaths, jaundice and exchange transfusion was asked in detail. Feeding history was documented.

Examination details included facial dysmorphism, skin for neurocutaneousstigmata, vital signs, weight, length, head circumference, tone reflexes, anterior fontanella, fundoscopy, hepato-spleenomegaly, cardiovascular and respiratory system examination.

First line investigations e.g. blood glucose, serum calcium, serum magnesium, serum sodium, and complete blood count with peripheral film and cerebrospinal fluid(CSF) for any evidence of infection, Serum urea / creatinine, liver function tests (LFT’s), cranial ultrasound (for intraventricular haemorrhage, edema, hydrocephalus or any malformation), electroencephalogram (EEG) were carried out in all patients. Second line investigations e.g. blood culture, CT scan (done in patients with bulging fontanella, focal neurological deficit and seizures resistant to antiepileptic drugs). MRI, screening for inborn error of metabolism, torch antibody titer (done in neonates with low birth weight, microcephaly, cataract, chorioretinitis, hepato-splenomegally and jaundice), reticulocyte count, coomb’s test and urine for reducing substances were done in selected cases guided by history, examination and initial investigations to reach the final diagnosis.

Hypoglycaemia was defined as blood glucose less than 40 mg/dl, hypocalcaemia as serum calcium less than 7.5 mg/dl. A hematocrit of less than 0.40 within the first week of life and 0.35 after the first week of life was regarded as severe anemia. CSF examination was considered abnormal when there were elevated CSF leukocytes, low CSF sugar (ratio of CSF to blood glucose less than 0.5), elevated CSF protein and/or positive smear for gram staining. 

In addition to correction of glucose, calcium and magnesium deficits, and anemia, seizures were routinely managed with injection diazepam (3-doses 5 mint apart) and in resistant cases loading dose of intravenous phenobarbitone 20 mg/kg, followed by phenobarbitone 5-8 mg/kg/day in two divided doses. Other supportive measures such as intravenous fluid and oxygen therapies were usually given according to the primary diagnoses. Fits not responding to above treatment had challenging dose of intravenous pyridoxine (100 mg). For tetanus neonatorum isolation, stimulus free environment, antibiotics, active and passive vaccination and more than one type of anticonvulsant were given.

The data was analyzed using SPSS-10.Descriptive statistics and frequencies were used for appropriate parameters.

## RESULTS

Neonatal seizures were more common in males (61.4%) than females (38.6%). There were 69.8% term and 30.2% preterm neonates. Majority of the neonatal seizures were seen in 1^st^ week life (80%). First day seizures were seen in 107 (37.5%) neonates. Birth asphyxia was the commonest cause of seizures as 53.7% followed by metabolic disturbance 17.5%, meningitis 10%, kernicterus 4.6%, intracranial hemorrhage 3.5%, tetanus 2.1%, drug withdrawal 2.1%, pyridoxine deficiency (detected on clinical response) 1.7%, and brain malformation 1.1%. Seizers with unknown etiology were present in 5.3% neonates.

**Table-I:** Etiological distribution of clinical neonatal seizures (n=285).

*Etiology*	*No. (%)*
Birth asphyxia	153 (53.7%)
Metabolic disturbance	50 (17.5%)
Meningitis	25 (10%)
Kernicterus	13 (4.6%)
Intracranial hemorrhage	10 (3.5%)
Tetanus	6 (2.1%)
Drug withdrawal	6 (2.1%)
Pyridoxine deficiency	5 (1.7%)
Brain malformation	3 (1.1%)

## DISCUSSION

Amongst neurological disorders, seizures are the most common in the neonates.^12^ The incidence noted by various studies range from 0.1–0.5% in term neonates^10^ and 10–23% in preterm neonates.^13^^,^^14^

Work done by Malik et al^6^, Park et al^14^ and Alcover et al^15^ showed that neonatal seizures were more prevalent in males. This is consistent with our study which showed that males were affected more as 61.4%.

Occurrence of seizures was found to be 80% in the first week of life in our study. Our findings are consistent as reported by other studies which showed that seizures were more common in the first 10 days of life.^6^^,^^16^ Neonatal seizures are more frequent in preterm babies^14^^,^^17^ which may be due to improved survival of premature babies in developed countries. We found seizures to be more common in term neonates (69.8%). This was also seen by other local studies conducted by Malik et al^6^ and Najeeb et al.^18^

Birth asphyxia is the commonest cause of neonatal seizures found globally.^10^^,^^12^^,^^19^ Our findings are in agreement with this. Most seizures in the asphyxiated new born occur in first 72 hours of life^6^^,^^7^^,^^15^ as in our study where 37.5% of the seizures occurred within 24 hours of life. Neonatal asphyxia is considered the predominant etiology in both premature and full term neonates.^16^ According to Volpe,^20^ the neonatal seizures are caused by hypoxic-ischemic encephalopathy in about 60% of neonates, independent of gestational age. Seizures in birth asphyxia may be due to hypoxic ischemic encephalopathy (HIE), transient metabolic disturbance or intracranial bleeding. Incidence of neonatal seizures can be reduced by reducing the risk factors responsible for birth asphyxia and by proper training of doctors and paramedical staff regarding delivery conductance and neonatal resuscitation.

Hypoglycemia,^13^^,^^15^ hypocalcaemia,^16^ hypomagnesemia^18^ and hypo/hypernatremiaare^19^ are important metabolic abnormalities causing neonatal seizures. Metabolic abnormalities were found in 17.5% of the cases in present study. Metabolic abnormalities may be due to prolong nothing per oral (NPO) or fasting. Exact mechanism of fits is unknown in hypoglycemia, hypocalcaemia and hypomagnesemia.^7^ These abnormalities of metabolic disturbance can be reduced by proper fluid and electrolyte management and in this way occurrence of seizures can be reduced.

Neonatal infections are found to be an important cause of neonatal seizures.^12^^,^^23^ Meningitis was noted in 10% of neonatal seizures cases in current study. Najeeb al^18^ also found 29% suffering from infections in a study conducted at Paediatric Department of Ayub Teaching Hospital Abbottabad. Neonatal infections can be reduced by proper sterilization, aseptic technique for any invasive procedure and proper selection and dose of antibiotics.

Kernicterus was present in 4.6% cases in our study whereas 4.5% were reported by Malik et al^6^ and 6% by another local study.^18^ Its incidence can be reduced by early detection, referral and prompt management (including phototherapy, exchange transfusion and drug therapy) of jaundiced newborn.

Birth trauma contributes to intracranial bleed and is an important cause of neonatal seizures.^24^ Good antenatal care and deliveries by an experienced person can reduce the incidence of birth trauma and hence intracranial bleed and neonatal seizures. Intracranial bleed was present in 3.5% cases as observed by others.^6^^,^^18^^,^^25^

Tetanus was present in 2.1% of neonates. Unvaccinated, poor sterilization and handling by Dai are the risk factors as observed by Ijaz et al.^26^ Risk factors for poor outcome after developing neonatal tetanus in a local study^18^ were found to be un-immunized mothers, rapid onset and shorter incubation period of disease. Incidence of tetanus can be reduced by eliminating the risk factors.

Pyridoxine deficiency was present 1.7% and detected clinically in the neonates when seizures were present inspite of correction of metabolic abnormalities, anticonvulsant therapy and absence of any identified cause. Seizures stopped clinically and by EEG study after single dose of 100mg iv pyridoxine. Level of serum pyridoxine was not done before iv pyridoxine because lifesaving is the first thing than study / research. So, pyridoxines may be added to the management in spite of the rare case of pyridoxine dependency, which may cause refractory seizures.

Brain malformations are involved rarely in neonatal seizures^21^^,^^27^ and found to be present in 1% of the cases in our study. The reported incidence of unknown etiology of clinical seizures varies from 2.4–5.3%.^28^ In our study, 5.3% cases were of presumed idiopathic fits while definite cause was present in all other remaining neonates suggesting that in contrast to older children neonatal seizures are rarely idiopathic.^10^ Since, the facilities for diagnosing in-born errors of metabolism are lacking at our setting and suggestive clinical features are not obvious, so it is very difficult to know the reasons for problems in inborn metabolism responsible for seizures of unknown etiology.

Neonatal seizures connected with transient metabolic abnormalities and the subarachnoid bleed have the best prognosis whereas birth asphyxia and brain abnormalities have the worst prognosis.^10^ Patients with meningitis have intermediate prognosis.^29^

## CONCLUSION

In most of the cases, the causes of neonatal seizures were present. Birth asphyxia was the main etiology identified in the majority of neonatal seizures. However, to establish the exact cause of seizures, more extensive work up is needed.

## References

[1] Mizrah EM (2001). Neonatal seizures and neonatal epileptic syndrome. Neurol Clin.

[2] Caravale B, Allemand F, Libenson MH (2003). Factors predictive of seizures and neurologic outcome in perinatal depression. Pediatr Neurol.

[3] Gebremariam A, Gutema Y, Leuel A, Fekadu H (2006). Early-onset neonatal seizures: types, risk factors and short-term outcome. Ann Trop Paediatr.

[4] Ballweg DD (1991). Neonatal seizures; an overview. Neonatal.

[5] Patrizi S, Holmes GL, Orzalesi M, Allemand F (2003). Neonatal seizures: characteristics of EEG Ictal activity in preterm and full term infants. Brain Dev.

[6] Malik BA, Butt MA, Shamoon M, Tehseen Z, Fatima A, Hashmat N (2005). Seizures etiology in the newborn period. J Coll Physicians Surg Pak.

[7] Sood A, Grover M, Sharma R (2003). Biochemical abnormalities in neonatal seizures. Indian J Pediatr.

[8] Upadhyay A, Aggarwal R, Deorari AK, Paul VK (2001). Seizures in the newborn. Indian J Pediatr.

[9] Laroia N (2000). Neonatal seizures. Indian Pediatr.

[10] Zupanc ML (2004). Neonatal seizures. Pediatr Clin North Am.

[11] Ballard JL, Khoury JC, Wedig K (1991). New Ballard Score, expanded to include extremely premature infants. J Pediatrics.

[12] Holanda MRR, Melo AN (2006). Comparative clinical study of preterm and full-term newborn neonatal seizures. Arq Neuropsiquiatr.

[13] Rennie JM, Bylan GB, David TJ (2002). Neonatal seizures. Recent Advances in Paediatrics 18.

[14] Park W, Kim DY, Jung CZ, Kim CD (1998). Clinical study of neonatal seizures. J Korean Child Neurol Soc.

[15] Alcover-Bloch E, Campistol J, Iriondo-Sanz M (2004). Neonatal seizures, our experience. Rev Neurol.

[16] Tekgul H, Gauvreau K, Soul J, Murphy L, Robertson R, Stewart J (2006). The Current Etiologic Profile and Neuro developmental outcome of Seizures in Term Newborn Infants. Pediatrics.

[17] Tharp BR (2002). Neonatal Seizures and syndromes. Epilepsia.

[18] Najeeb S, Qureshi AM, Rehman A, Ahmad F, Shah S, Khan AY (2012). Aetiology and types of neonatal seizures presenting at Ayub Teaching Hospital Abbottabad. J Ayub Med Coll Abbottabad.

[19] Verrotti A, Latini G, Cicioni P, Felice CD (2004). New trends in neonatal seizures. J Pediatr Neurol.

[20] Volpe JJ (2001). Neonatal seizures. Neurology of the Newborn.

[21] Jin S, Hahn MD, Donald M, Olson MD (2004). Etiology of neonatal seizures. Neo Reviews.

[22] Nordli DR Jr, De Vivo DC (2002). Classification of infantile seizures, implications for identification and treatment of inborn error of metabolism. J Child Neurol.

[23] Rufo-Campos M, Gonzalez Meneses-Lopez A, Rangel-Pineda C (2000). Cerebral seizures in neonatal period: semeiology, evolution and factors of influence. Rev Neurol.

[24] Pollina J, Dias MS, Li VK, Kachurek D, Arbesman M (2001). Cranial birth injuries in term newborn infants. Pediatr Neurosurg.

[25] Ajay K, Ashish G, Bibek T (2007). Clinico-etiological and EEG profile of neonatal seizures. Indian J Pediatr.

[26] Ijaz I, Khan HI (2000). Risk factors of neonatal Tetanus. Pak Pediatr J.

[27] Barkovich AJ, Kuzniecky RI, Jackson MD, Guerrini R, Dobyns WB (2001). Classification system for malformations of cortical development. Neurology.

[28] Mizrahi EM, Kellaway P (1998). Diagnosis and managment of neonatal seizures.

[29] Rennie JM, Boylan GB (2003). Neonatal seizures and their treatment. Curr Opin Neurol.

